# Two‐parametric prescan calibration of gradient‐induced sampling errors for rosette MRI

**DOI:** 10.1002/mrm.30355

**Published:** 2024-10-22

**Authors:** Peter Latta, Radovan Jiřík, Jiří Vitouš, Ondřej Macíček, Lubomír Vojtíšek, Ivan Rektor, Michal Standara, Jan Křístek, Zenon Starčuk

**Affiliations:** ^1^ Central European Institute of Technology Masaryk University Brno Czech Republic; ^2^ Institute of Scientific Instruments of the Czech Academy of Sciences Brno Czech Republic; ^3^ Department of Radiology Masaryk Memorial Cancer Institute Brno Czech Republic

**Keywords:** gradient imperfections, k‐space misalignment, rosette trajectory, trajectory estimation

## Abstract

**Purpose:**

The aim of this study was to develop a simple, robust, and easy‐to‐use calibration procedure for correcting misalignments in rosette MRI k‐space sampling, with the objective of producing images with minimal artifacts.

**Methods:**

Quick automatic calibration scans were proposed for the beginning of the measurement to collect information on the time course of the rosette acquisition trajectory. A two‐parameter model was devised to match the measured time‐varying readout gradient delays and approximate the actual rosette sampling trajectory. The proposed calibration approach was implemented, and performance assessment was conducted on both phantoms and human subjects.

**Results:**

The fidelity of phantom and in vivo images exhibited significant improvement compared with uncorrected rosette data. The two‐parameter calibration approach also demonstrated enhanced precision and reliability, as evidenced by quantitative $T_{2}^{*}$ relaxometry analyses.

**Conclusion:**

Adequate correction of data sampling is a crucial step in rosette MRI. The presented experimental results underscore the robustness, ease of implementation, and suitability for routine experimental use of the proposed two‐parameter rosette trajectory calibration approach.

## 1 INTRODUCTION

Since the introduction of the rosette acquisition in the late 1990s,^1^ it has consistently attracted attention due to its unique spectral properties and specific sampling pattern. Over the last two decades, this technique has been used in various studies to demonstrate these features and characteristics.

Rosette techniques find utility in various applications, such as spectroscopic imaging for metabolite quantification in the brain,^2^, ^3^ T_1_/T_2_‐weighted myocardial tissue characterization with simultaneous water–fat separation,^4^, ^5^ or the recently proposed quantification of the short $T_{2}^{*}$ signal components in the brain using 3D dual‐echo rosette acquisition.^6^ In general, rosette MRI poses many attractive features, including robustness to motion and inhomogeneity self‐correction, as B_0_ maps can be calculated from the actual measured data and off‐resonance behavior, which leads to signal loss rather than blurring.^1^ Rosette‐based acquisition‐sampling schemes have also proven to be well‐suited for combination with iterative reconstruction techniques, such as compressed‐sensing applications.^5^, ^7^, ^8^

In general, all non‐Cartesian techniques are prone to artifacts caused by gradient imperfections and delays, which are responsible for discrepancies between prescribed and actual acquisition trajectories, which propagate into subsequent degradation of image quality. This necessitates the development of various strategies to compensate for those sampling deviations, which should be incorporated into the image acquisition and reconstruction scheme. The compensation takes two steps: measurement and correction. The first step involves measuring and characterizing the gradient system using dedicated gradient field probes,^9^, ^10^ monitoring currents at the gradient amplifier outputs,^11^ execution of calibration scans,^12^ or extracting this information directly from the image data.^13^, ^14^ The second step is correction for these imperfections, which can be done either directly during data acquisition^15^, ^16^ or, more commonly, during data reconstruction.^17^

Although most studies using rosette acquisition do not provide detailed information about the applied trajectory correction, previously published works include examples of estimating readout gradient delays using separate calibration measurements or obtaining them directly from measured image data. For example, in one case, the optimal gradient delays were empirically estimated by performing an extensive search for the best match of $T_{2}^{*}$ fit between Cartesian and rosette data of a ferumoxytol phantom.^18^ In another study, gradient delays were determined directly from image data using a modified technique previously applied for radial acquisition^19^ or estimating gradient delays from the misalignment of the k‐space origin crossing of multishot rosette trajectories.^20^

This study aims to develop a simple calibration method for accurately determining gradient delays, seamlessly integrated into rosette MRI experiments without requiring separate exams. The proposed rosette trajectory correction includes two steps: (i) quick automatic calibration scans performed at the beginning of the measurement, inspired by the previously suggested spiral imaging calibration technique that was adapted for rosette‐specific requirements,^21^ and (2) fitting the calibration data with a two‐parametric rosette trajectory model to estimate the actual trajectory. The proposed methodology was verified and tested using both phantom and in vivo experiments. Performance and data fidelity were also scrutinized through a comparison of $T_{2}^{*}$ values obtained from multi‐echo rosette and Cartesian images.

## 2 METHODS

### 2.1 Approximation of experimental rosette trajectory

The rosette trajectory can be described as follows^1^, ^22^:

(1) $$k\left(t\right)=k_{\max}\sin\left(\omega_{1}t\right)\;e^{{i\omega }_{2}t}$$

where $\omega_{1}$ determines the oscillations in the radial direction across the k‐space origin; $\omega_{2}$ controls the rotation speed of the radial trajectory around the axial direction; and i denotes the imaginary unit $\sqrt{-1}$. For further clarification, it is important to note that Eq. (1) is related to the logical coordinate system, with the real part corresponding to the readout direction and the imaginary part corresponding to the phase encoding direction. The corresponding gradient waveform to generate such trajectory can be expressed as follows^1^:

(2) $$\begin{array}{ll} g\left(t\right) & =\frac{2\pi }{\gamma }\;\frac{\mathrm{dk}\left(t\right)}{\mathrm{dt}}\\ & =\frac{2\pi }{\gamma }k_{\max}\left({i\omega }_{2}\sin\left(\omega_{1}t\right)+\omega_{1}\cos\left(\omega_{1}t\right)\right)\;e^{{i\omega }_{2}t} \end{array}$$

where $\gamma =2.675\cdot 10^{8}\;\mathrm{rad}/\left(s\cdot T\right)$ denotes the gyromagnetic ratio. By separating the real and imaginary parts (corresponding to the frequency and phase encoding directions of the Cartesian k‐space sampling), the previous equation can be further rearranged into two orthogonal components, as follows:

(3) gRe(t)=πγkmaxω1−ω2cosω1−ω2t+ω1+ω2cosω1+ω2tgIm(t)=πγkmax−ω1−ω2sinω1−ω2t+ω1+ω2sinω1+ω2t

Equation (3) suggests that the rosette readout gradient waveform consists of two frequency components given by the following relations:

(4) $$\begin{array}{c} f_{L}=\frac{1}{2\pi }\;\left(\omega_{1}-\omega_{2}\right)\\ f_{H}=\frac{1}{2\pi }\;\left(\omega_{1}+\omega_{2}\right) \end{array}$$

where $f_{L}$ and $f_{H}$ are the lower and higher frequency components of the rosette gradient waveform, respectively. The output gradient waveform is affected by the properties of the physical gradient channel, which can be characterized in the frequency domain by the gradient system transfer function (GSTF),^23^ specific for each of the x‐, y‐ and z‐channels of the gradient system. In those cases, in which information about the GSTF is available, the actual spectrum of the gradient waveform can be computed as

(5) $$G^{\mathrm{act}}\;\left(f\right)=G\left(f\right)\cdot H\left(f\right)$$

where H(f) is the gradient channel's GSTF; G(f) is the frequency spectrum of the prescribed gradient waveform; and $G^{\mathrm{act}}\;\left(f\right)$ is the frequency spectrum of the actual magnetic gradient field acting on the sample. The GSTF acts on the requested gradient waveform spectrum by affecting its amplitude and phase. For modeling amplitude alterations in the rosette gradient spectrum, we introduced the parameter R, which is defined as the GSTF magnitude ratio of the higher and lower frequencies, as follows:

(6) $$R=\frac{\left|H\left(\omega_{H}\right)\right|}{\left|H\left(\omega_{L}\right)\right|}$$

The parameter R reflects the ability of the gradient system to reproduce the amplitude of both frequency components and can differ from Value 1, depending on the frequency attenuation factor. Moreover, the GSTF phase typically exhibits linear phase characteristics, especially in the range of up to a few kHz. This linear phase response is linked with time‐domain shifts and is observed as a gradient waveform time delay, $t_{d}$. We want to emphasize that the two‐parameter model more accurately represents the complex nature of the GSTF than a model based solely on delay. This approach allows for addressing both GSTF components: phase and amplitude. Although the time‐delay parameter t_d_ captures the phase characteristics, the additional parameter R targets the amplitude properties. Therefore, accounting for the amplitude ratio, R, and the time delay difference, $t_{d}$, the actual rosette gradient waveform can be expressed as follows:

(7) gReactt,td,R=πγkmaxω1−ω2cosω1−ω2t−td+R⋅ω1+ω2cosω1+ω2t−tdgImactt,td,R=πγkmax−ω1−ω2sinω1−ω2t−td+R⋅ω1+ω2sinω1+ω2t−td

and the corresponding orthogonal components of the k‐space trajectory are given as

(8) kReactt,td,R=12⋅kmax−sinω2−ω1t−td+Rsinω2+ω1t−tdkImactt,td,R=12⋅kmax−cosω2−ω1t−td+Rcosω2+ω1t−td

Equation (8) represents a two‐parametric model of the actual rosette k‐space trajectory affected by a gradient system delay $t_{d}$ and an amplitude attenuation parameter R.

### 2.2 Rosette trajectory estimation

The estimation of rosette trajectories was designed as a two‐stage procedure. In the first step, trajectory calibration measurements were conducted so that k‐space center crossings along the rosette data sampling trajectory can be determined. This was followed by the second step, in which the measured crossing delays served as input data allowing estimation of parameters $t_{d}$ and R for each physical gradient‐system channel using the mathematical model based on Eq. (8). Our rosette trajectory calibration was performed using an adapted technique previously proposed for measuring time delays in spiral MRI.^21^ In short, gradient channel delays were calibrated using two measurements with opposite gradient polarities, allowing precise detection of the k‐space center intersection by the acquisition trajectory. The exact intersection time was determined by cross‐correlating both recorded signals and calculating the gradient delays from the time differences between the theoretical and actual crossings (for more details, see Figure 1 in Robison et al.^21^). We applied this technique for rosette trajectory calibration, introducing two modifications. The first modification acknowledges that the rosette trajectory is achieved by gradient pulses in two orthogonal directions of the logical coordinate system (here corresponding to the real and imaginary components of k‐space). In addition, the rotation of successive rosettes by the golden‐angle ratio means rotation of this logical coordinate system around its origin. Furthermore, for an oblique tilt of the imaging plane, the gradient pulses consist of components played out simultaneously along all physical axes. To ensure better precision, both rosette trajectory components (i.e., real and imaginary in Eq. [1]) were used in the calibration measurements, resulting in a total of 12 calibration scans (2 orthogonal rosette components × 2 gradient polarities × 3 gradient physical channels). The second modification pertains to the selection of only a certain number of the k‐space center crossings, as not all intersections are suitable for calibration. Only those crossings producing well‐defined sharp signal peaks were selected that ensured reliable and precise detection of the trajectory delays, which is typical for the k‐space center passages occurring with high gradient amplitude values. To meet this requirement, we manually preselected particular transitions for each specific rosette trajectory type (determined by parameters n_1_ and n_2_ ^1^), which were later used automatically in the calibration measurements.

**FIGURE 1:**
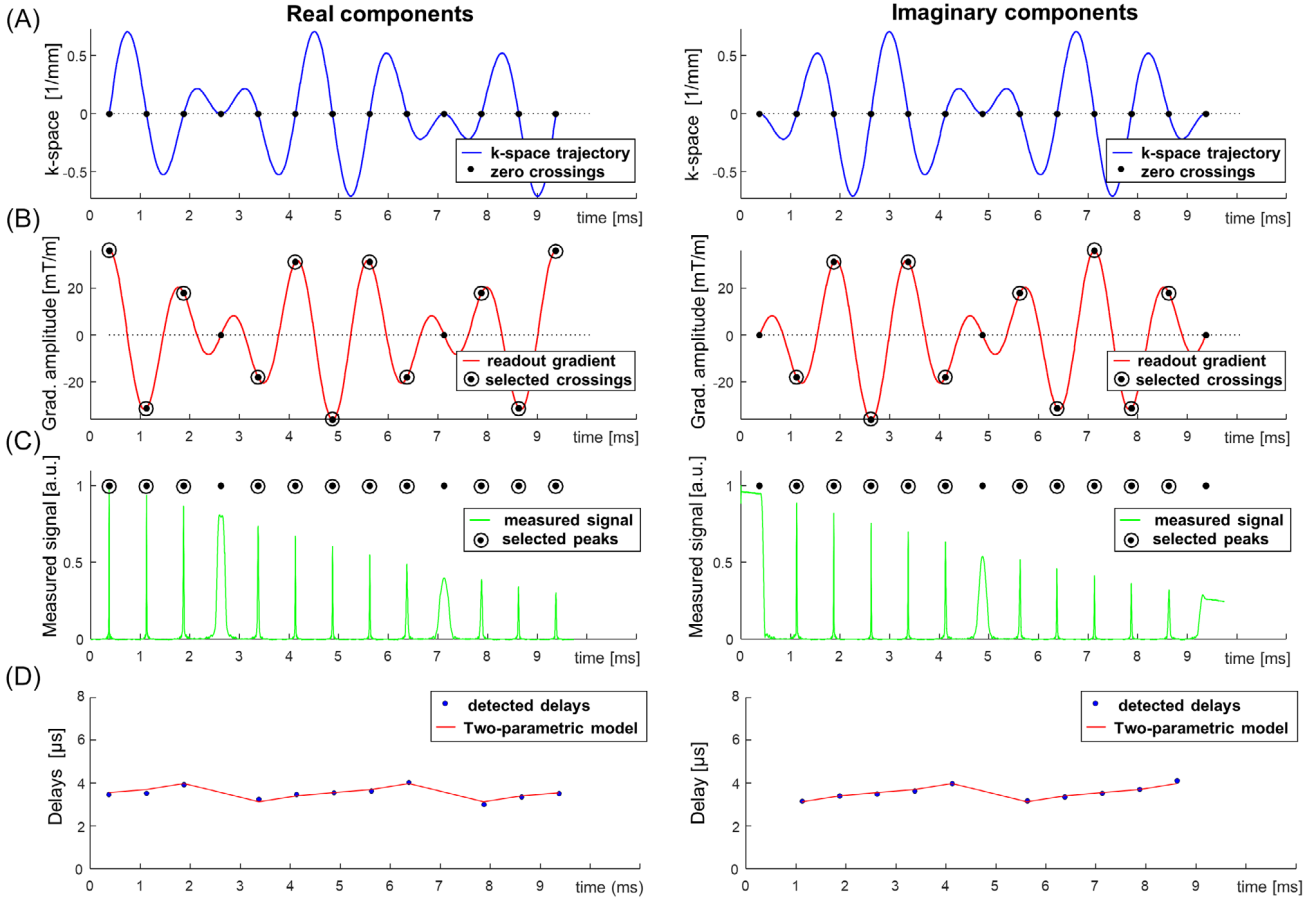
Example of the calibration procedure for the 12‐petal rosette trajectory Type 6/1, as obtained on the phantom for the x‐channel of the gradient system. (A–C) The crossings of the acquisition trajectory with the k‐space origin are indicated by dots (A), with only those occurring with sufficiently high gradient amplitudes (B) selected for trajectory calibration (*encircled dots*) with measured signal amplitude shown in (C). (D) The detected delays are fitted by a “two‐parametric correction” (*red line*).

In the second step, we approximated the actual trajectory with a mathematical model, using the following objective function in the search for parameters $t_{d}$ and R (performed separately for each physical gradient‐system channel):

(9) Ftd,R=∑m=1M∆tRe(m)−DRem,td,R+∑n=1N∆tIm(n)−DImn,td,R

where M and N represent the total number of preselected calibration points; ∆tRe(m),∆tIm(n) denote the *m*th and *n*th measured calibration delays of the zero‐crossing real and imaginary trajectory components, respectively; and DRe, DIm are operators predicting the *m*th and *n*th crossing delays for the current estimates of $t_{d}$ and R, based on the mathematical model (Eq. [8]). The searched parameters are determined by minimizing the objective function (i.e., $\min_{t_{d},R\in R}\left(F\left(t_{d},R\right)\right)$ and used subsequently for estimation of the actual sampling trajectory during image reconstruction.

Because the calibration and image data are associated with different coordinate systems, namely physical and logical, respectively, the trajectory correction procedure must adhere to the approach previously suggested.^24^, ^25^ First, the theoretical image sampling trajectories were transformed from the logical to the physical coordinate system using a rotation matrix. Second, based on the gradient channels' calibration measurements, calculations of the actual physical trajectories were carried out. It should be noted that the calibration procedure will yield three pairs of constants ($t_{d}$ and R) as output, with one pair for each physical gradient channel. Finally, the calculated physical rosette trajectories were transformed back into the image coordinate system, and the estimated logical trajectories were further used for image reconstruction with the corrected k‐space trajectory.

### 2.3 Experimental setup

The experiments were conducted using a clinical whole‐body 3T MRI system (Magnetom Prisma; Siemens Medical Solutions, Erlangen, Germany). The gradient system of the instrument can deliver a maximum gradient strength of 80 mT/m with a simultaneous slew rate of 200 Tm^−1^ s^−1^. The experimental setup involved a whole‐body RF coil for transmitting, while receiving was carried out using an integrated spine matrix coil combined with either a four‐channel or 18‐channel flexible set of phase array coils for measurements on phantoms or volunteers, respectively.

For this study, a custom 3D stack of rosettes pulse sequence was developed. This sequence uses a combination of Cartesian phase encoding along the slab‐selection direction and rosette trajectory sampling for the in‐plane dimensions. The rosette readouts used prephasing gradient lobes, which addressed the issue of infinite slew rate at the beginning of the gradient.^26^

All data in this study were acquired using 12‐petal rosette trajectories, prescribed with six oscillation cycles (n_1_ = 6) and combined with three different numbers of rotations (n_2_ = 1, 5, and 7), further referred to in the form n_1_/n_2_ (i.e., as rosette Types 6/1, 6/5, and 6/7). Therefore, the correction method was demonstrated for both classes of rosette trajectories: Class I (ω_1_ > ω_2_) and Class II (ω_1_ < ω_2_).^1^, ^7^

Each sequence repetition began with slab‐selective RF excitation, followed by either trajectory calibration or image scans (see Figure S1). The calibration scans were conducted at the beginning of the sequence, using a fixed TR of 300 ms, resulting in a total duration of approximately 3.6 s, after which the image data acquisition commenced.

As mentioned earlier, the k‐space trajectory calibration procedure relies on careful selection of the calibration time points, preferably located within narrow signal peak areas where the readout gradient waveform exhibits high amplitude values. This ensures that the k‐space center transitions are rapid, allowing reliable detection and accurate estimation of the readout gradient delays. The selection of calibration time points used for the purposes of this study is summarized in Table 1.

**TABLE 1:** Summary of calibration time points selection, determined using the higher of both frequencies (i.e., oscillation frequency $\omega_{1}$ in Class I trajectories and rotational frequency $\omega_{2}$ in Class II trajectories).

Rosette trajectory Type 6/1: tRe(n)=tIm(n)=nπω1,n=0,1,2,…,2n1															
n	0	1	2	3	4	5	6	7	8	9	110	11	12		
Re	⨀	⨀	⨀	•	⨀	⨀	⨀	⨀	⨀	•	⨀	⨀	⨀		
Im	•	⨀	⨀	⨀	⨀	⨀	•	⨀	⨀	⨀	⨀	⨀	•		

Rosette trajectory Type 6/5: tRe(n)=tIm(n)=nπω1,n=0,1,2,…,2n1															
*n*	0	1	2	3	4	5	6	7	8	9	110	11	12		
Re	⨀	⨀	⨀	⨀	⨀	⨀	⨀	⨀	⨀	⨀	⨀	⨀	⨀		
Im	⨀	⨀	⨀	⨀	⨀	⨀	⨀	⨀	⨀	⨀	⨀	⨀	⨀		

Rosette trajectory Type 6/7: tRe(n)=nπω2,tIm(n)=(n+0.5)πω2,n=1,2,…,2n2															
*n*	0	1	2	3	4	5	6	7	8	9	110	11	12	13	14
Re	⨀	⨀	⨀	•	⨀	⨀	⨀	⨀	⨀	⨀	•	⨀	⨀	⨀	•
Im	•	⨀	⨀	⨀	⨀	⨀	⨀	•	⨀	⨀	⨀	⨀	⨀	⨀	•

*Note*: Only the time points where narrow signal peaks formed were selected, indicated by the symbol ⨀ (see Figure 1 and Figures S2).

After the completion of each phase‐encoding cycle, the rosette interleaves were incrementally rotated by a golden ratio–related angle, which provided reconstruction flexibility by allowing retrospective selection of the number of acquisition shots per image frame and making it possible to use iterative reconstruction algorithms. For the 12‐petal rosette trajectory, there is a 30° angle between neighboring petals. Therefore, the rotation increment was calculated as 30°/GR ≈ 18.5410^0^, where GR denotes the golden ratio.^27^ We found that this approach helps to achieve an evenly distributed rosette sampling trajectory (see Figure S2).

The data processing and image reconstruction were conducted offline using custom‐developed routines written in *MATLAB* (The MathWorks, Natick, MA, USA). The parameters $t_{d}$ and R were determined by minimizing the objective function (Eq. [9]) using the *MATLAB* fminsearch routine with a Nelder–Mead simplex direct search algorithm. The rosette images were reconstructed using a Kaiser‐Bessel interpolation kernel (width = 3 and *β* = 13.9068) and density compensation based on the Voronoi diagram (using the *MATLAB* Voronoi routine). In the case of $T_{2}^{*}$ mapping, the multi‐echo data were constructed by segmenting the rosette flower into six temporal segments, each containing two consecutive petals, and grouping corresponding sections across shots.

The rosette reconstruction was conducted for comparison purposes with three types of trajectory corrections. The corresponding data sets will be further referred to as “no correction,” “delay correction,” and “two‐parametric correction.” The “no correction” rosette data sets were reconstructed using the nominal trajectory. The “delay correction” data sets used trajectory correction calculated as the average value of the calibration delays. Finally, the “two‐parametric correction” data sets were based on a rosette trajectory calculated using Eq. (8).

#### 2.3.1 B_0_ inhomogeneity correction

All presented data underwent a B_0_ inhomogeneity correction, which was carried out in two steps. First, the B_0_ map was derived from the rosette data following a previously proposed approach.^4^ After computing the B_0_ map, the actual rosette data were corrected using an acquisition time segmentation strategy.^28^ In our implementation, all k‐space data were divided into successive time segments of half petal length. For example, for a 12‐petal rosette trajectory, the sampled data were divided into 24 subsets, and each subset was then used in the previously described image reconstruction. The final corrected image was then calculated by summing images of all subsets according to the following relationship:

(10) $$I_{\text{corr}}=\sum_{m=1}^{M}I_{m}e^{-i\;2\pi \;{\Delta B}_{0}\;\left(m-1\right)∆T_{\mathrm{seg}}}$$

where $I_{\text{corr}}$ represents the corrected complex image; $I_{m}$ is the complex image reconstructed from the mth data segment; M is the total number of segments; $∆T_{\mathrm{seg}}$ is the segment duration; and ${\Delta B}_{0}$ represents the resonance frequency inhomogeneity map calculated according Eq. (2) in Liu et al.^4^

#### 2.3.2 Phantom study

The performance of the proposed rosette trajectory correction scheme was first assessed using a structural phantom comprised of one central cylinder (inner diameter = 140 mm) with two smaller ones (inner diameter = 60 mm) positioned on opposite sides. All vessels were filled with a MnCl_2_ solution with a concentration of 0.5625 mM (T_1_ ˜ 278 ms, T_2_ ˜ 16 ms). The phantom was positioned quasi‐parallel to the physical z‐axis with a slight rotation around the x‐axis (˜16°), applying a similar slice orientation as in the volunteer pelvic studies conducted later.

Rosette data were collected using the following acquisition parameters: FOV = 350 mm, matrix size = 256 × 256, 24 slices with a 3‐mm slice thickness, TR = 25 ms, flip angle = 24°, and 128 golden‐ratio angle increments, resulting in a total experimental time of 77 s. The experiment was repeated with all three implemented rosette trajectories using the following gradient amplitudes, slew rates, and readout bandwidths: Rosette 6/1: G_max_ = 33.32 mT/m, S_max_ = 132.8 T/m/s, bandwidth (BW) = 500 kHz; Rosette 6/5: G_max_ = 26.99 mT/m, S_max_ = 143.6 T/m/s, BW = 384.615 kHz; and Rosette 6/7: G_max_ = 22.49 mT/m, S_max_ = 139.0 T/m/s, BW = 333.333 kHz. Additionally, Cartesian 3D gradient‐echo reference images were acquired with parameters corresponding to those used in the rosette acquisition.

#### 2.3.3 Phantom $T_{2}^{*}$ relaxation study

A phantom containing seven test tubes filled with graded concentrations of MnCl_2_ (0.28, 0.56, 1.125, 1.5, 2.25, 3.0, and 4.5 mM) was constructed for relaxation study purposes. The test tubes were oriented parallel to the z‐axis, and rosette data were acquired with the following parameters: FOV = 240 mm, matrix size = 256 × 256, single slice at 5‐mm thickness, TR = 30 ms, flip angle = 23^º^, using 64 rosette shots, for a total experimental time of 1.9 s. All rosette acquisitions used identical gradient amplitude of G_max_ = 19.68 mT/m and were combined with slew rates S_max_ = 31.8, 52.4, and 73.0 T/m/s for trajectory Types 6/1, 6/5, and 6/7, respectively. All acquisitions used an identical readout BW = 238.10 kHz. Six multi‐echo images (TE = 3.35, 7.48, 11.61, 15.74, 19.88, and 24.01 ms) were reconstructed by grouping each two successive petals for $T_{2}^{*}$ fitting. Cartesian multiple gradient‐echo (TE = 1.86, 5.68, 10.80, 15.92, 21.04, and 26.16 ms) reference data were acquired for comparison as well.

For calculation of $T_{2}^{*}$ values, a square region of interest was manually positioned into the center of each vial and stored so that all data sets could be perfectly coregistered (see Figure S4). The $T_{2}^{*}$ analyses were performed in *MATLAB* using a nonlinear least‐squares fitting of the multi‐echo magnitude data. A Bland–Altman analysis was applied for the evaluation of agreement between rosette and reference data. A Wilcoxon signed‐rank test was applied for comparison of the uncorrected and corrected data; cases with *p*‐values lower than 0.05 were considered statistically significant.

#### 2.3.4 Volunteer study

A group of 5 healthy volunteers underwent scanning, and performance of the two‐parametric k‐space trajectory correction method was evaluated for an in vivo pelvis examination. Institutional approvals were obtained before the in vivo study, and the participants provided a written, informed consent before the examinations. Initially, quick pilot scans were obtained to precisely position a 3D slab over the prostatic urethra, with a slight tilt applied perpendicular to the dorsal prostate contour, in accordance with the diagnostic protocol described previously.^29^ Specifically, the original slab orientation, with readouts in the axial plane, was rotated by a few degrees (typically between 10° and 20°) around the physical x‐axis toward the coronal plane. The slab positioning was followed by a standard shimming procedure to optimize B_0_ homogeneity and reduce off‐resonance effects within the selected volume. All in vivo rosette acquisitions were performed with an identical FOV of 240 mm, a matrix size of 256 × 256, and 256 golden ratio–angle increments. The rest of the parameters were chosen to satisfy the limits of the peripheral nerve stimulation for each rosette trajectory type as follows: Rosette 6/1 used TR = 16 ms, flip angle = 230°, G_max_ = 40.99 mT/m, S_max_ = 137.9 T/m/s, BW = 416.67 kHz, readout time of 12.3 ms, with a total acquisition period of 98.3 s. Rosette 6/5 used TR = 20 ms, flip angle = 250°, G_max_ = 31.48 mT/m, S_max_ = 134.1 T/m/s, BW = 312.5 kHz, readout time of 15.8 ms, with a total acquisition period of 122.8 s. Rosette 6/7 used TR = 22 ms, flip angle = 260°, G_max_ = 26.59 mT/m, S_max_ = 133.3 T/m/s, BW = 263.16 kHz, readout time of 18.6 ms, with a total acquisition period of 136 s.

A modulation of the rosette k‐space signals by 440 Hz was applied to produce fat images.^4^ In the case of composite water–fat images, both the original and the modulated signals were added together. Two trained radiologists, each with over 20 years of experience, independently assessed the images in terms of artifact‐level distortion and overall image quality using a scale as previously suggested for artifact rating in breast studies.^30^

## 3 RESULTS

### 3.1 Phantom study

An example of the rosette Type 6/1 trajectory calibration performed during the phantom imaging experiment is depicted in Figure 1. The delays detected over the course of the rosette trajectory were not constant; however, they exhibited a specific “zigzag” pattern (Figure 1D), which can be fitted with the two‐parametric model of Eq. (9). Additional examples of calibration procedures applied for the case of rosette trajectory Types 6/5 and 6/7 are provided in Figure S3.

**FIGURE 2:**
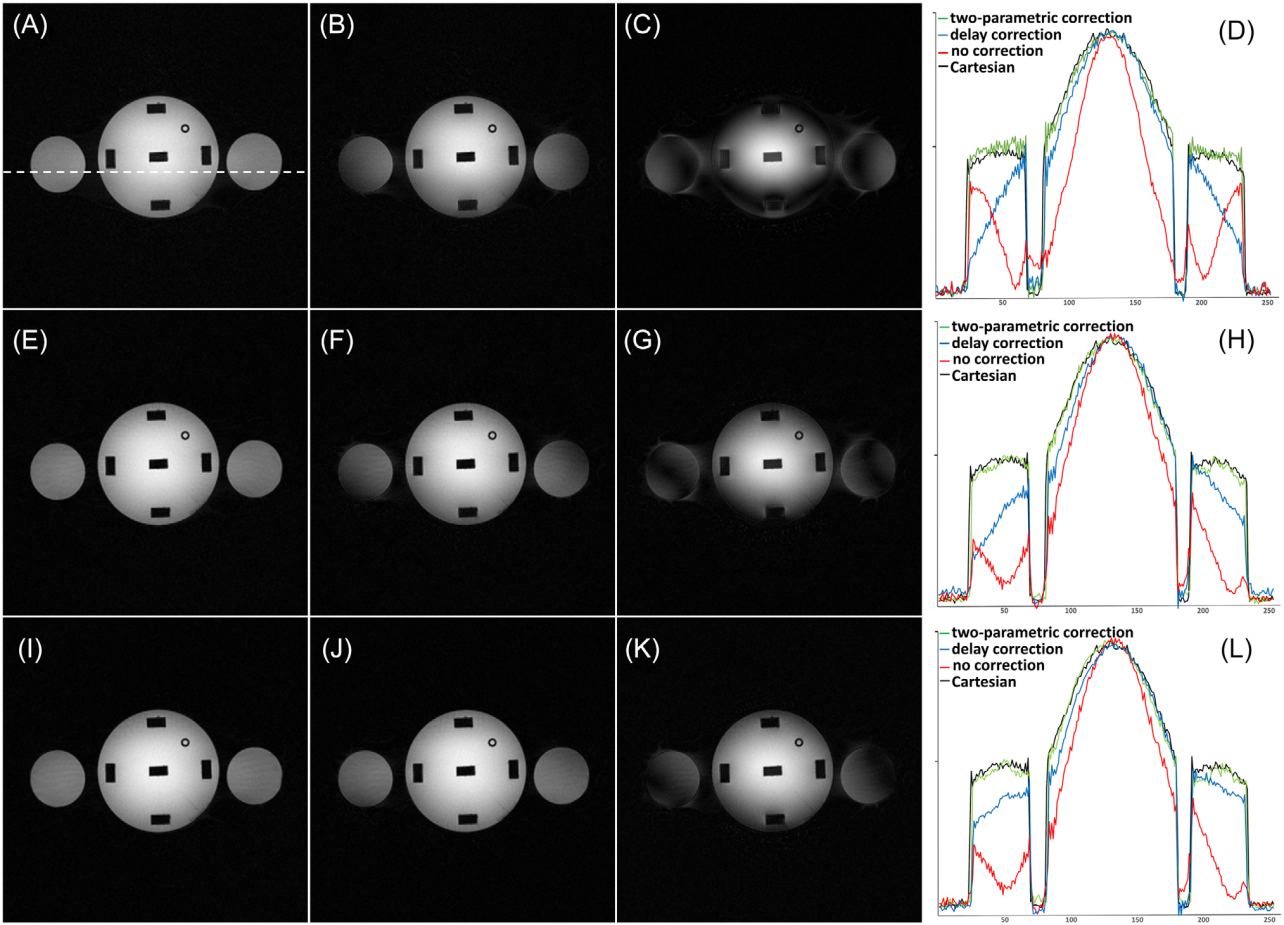
Phantom images acquired with the 12‐petal rosette trajectories: Type 6/1 (A–C), Type 6/5 (E–G), and Type 6/7 (I–K). The images in the left column (A,E,I) were reconstructed using the “two‐parametric correction” method, whereas images in the middle column (B,F,J) were reconstructed using “delay correction,” and images in the right column (C,G,K) were reconstructed without any correction. Profiles plotted along the dotted line for rosette and reference Cartesian images are shown for comparison: Type 6/1 (D), Type 6/5 (H), and Type 6/7 rosette trajectory (L).

The impact of the trajectory calibration on image fidelity is demonstrated in Figure 2, where the “two‐parametric correction” rosette data are compared with both “no correction” and “delay correction” images. The differences in images obtained with no correction and the two types of corrections can clearly be depicted in the one‐dimensional intensity plots, where the Cartesian FLASH data are provided for reference as well (Figure 2D,H,L).

A summary of the parameters determined by the rosette trajectory calibration procedures is listed in Table S1.

### 3.2 Phantom $T_{2}^{*}$ relaxation study

**FIGURE 3:**
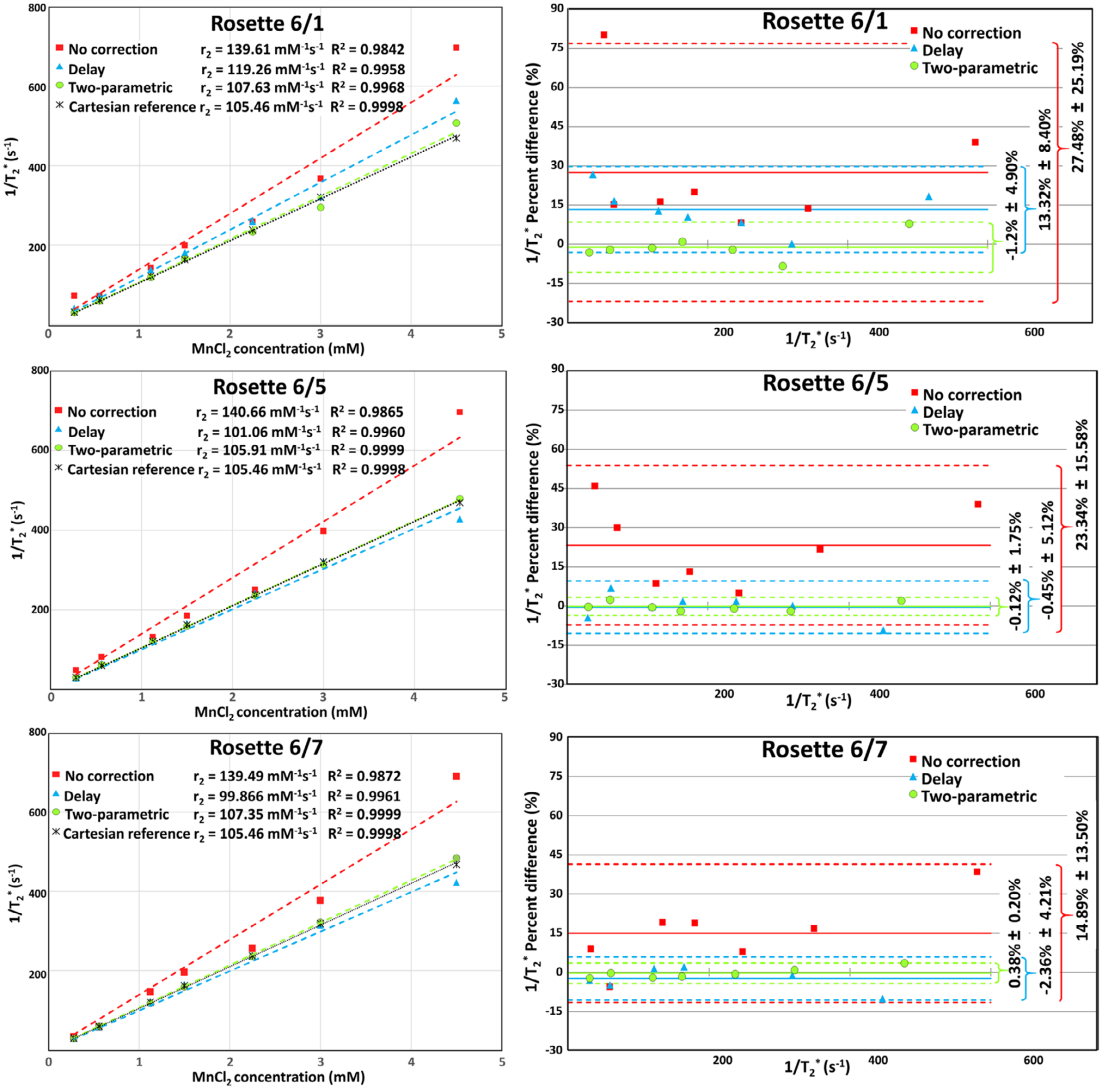
Relaxation rate measurements with the MnCl_2_ phantom and all three rosette trajectory types (*left column*). Although both “no correction” and “delay correction” data exhibit a strong linear trend with NnCl_2_ concentration changes (coefficient of determination R^2^ > 0.98), a systematic bias to the reference Cartesian data can be noticed. Bland–Altman plots (*right column*) of 1/$T_{2}^{*}$ values were used to compare the correction methods. The solid line shows the average difference between the rosettes and the reference data, whereas the dashed lines indicate the 95% upper and lower limits of agreement, respectively (exact values are indicated by curly brackets on the right side of the plots).

Figure 3 demonstrates the influence of the rosette trajectory correction on $T_{2}^{*}$ relaxometry precision performed using the MnCl_2_ phantom. The fitted values of the relaxation rates versus MnCl_2_ concentrations are shown as obtained for all three rosette trajectory types (Figure 3, left column). The reference relaxation rates versus concentrations were determined from the Cartesian measurements performed with the MnCl_2_ phantom and resulted in the reference relaxivity r_2_ = 105.46 mM^−1^ s^−1^ (R^2^ = 0.9998). The relaxation rates obtained from the rosette measurements without trajectory correction exhibit more than a 32% bias for all three rosette trajectory types when compared with the reference data. This was improved by performing trajectory calibration with the “delay correction,” resulting in a reduction of relative error to 13.08% for rosette trajectory 6/1 and less than 6% for both 6/5 and 5/7 trajectory types. The accuracy was further improved by performing trajectory calibration with the “two‐parametric correction,” resulting in a reduction of the relative error to less than 2% for all three rosette trajectory types.

The Bland–Altman 1/$T_{2}^{*}$ plots (Figure 3, right column) revealed mean differences of 27.48%, 23.34%, and 14.89% between the uncorrected‐rosette relaxation rates and the corresponding reference values for the 6/1, 6/5, and 6/7 rosette trajectory types, respectively. The delay‐calibration correction showed failure in the case of the rosette Type 6/1, as the mean difference dropped only to 13.32%, and no significant difference (*p* = 0.07813) was achieved when compared with analyses using uncorrected data. However, for rosette 6/5 and 6/7 types, the delay‐corrected 1/$T_{2}^{*}$ data exhibited only a −0.45% and −2.36% mean difference to the reference, both with significant differences (*p* = 0.01563 and *p* = 0.03125) compared with uncorrected data. The best results were achieved using the two‐parametric correction, achieving mean differences of −1.2%, −0.12%, and 0.38% with significantly higher differences from the reference than obtained for the uncorrected rosette data sets (*p* = 0.01563, *p* = 0.01563, and *p* = 0.03125) for all three rosette trajectory types.

### 3.3 Volunteer study

**FIGURE 4:**
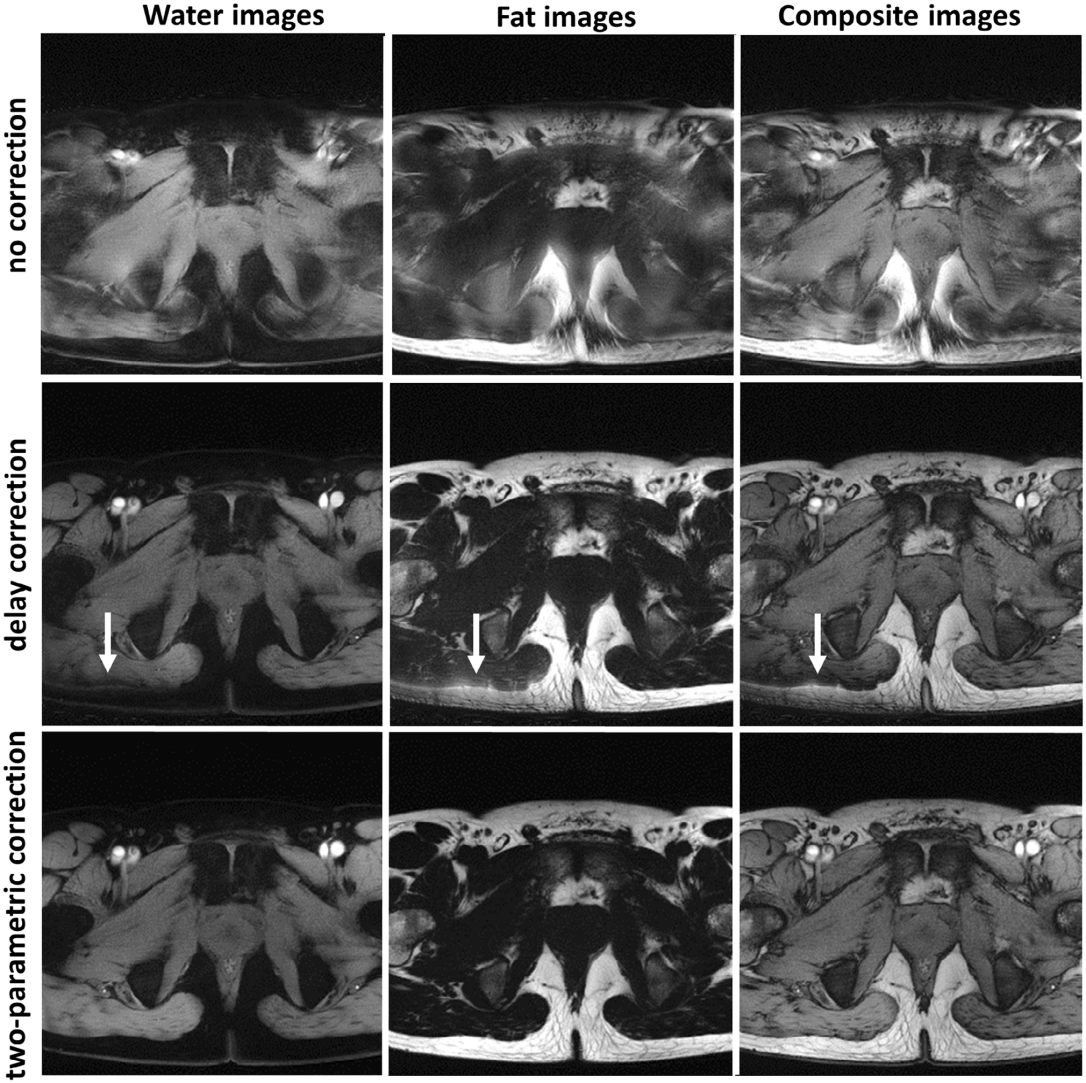
Example of in vivo pelvis rosette images (trajectory Type 6/5) reconstructed with “no correction” (*top row*), with “delay correction” (*middle row*), and with “two‐parametric correction” (*bottom row*). The left column shows water images, the middle column shows fat images, and the right column shows water/fat composite images. Analogous images acquired with trajectory Types 6/1 and 6/7 are available in Figures S5 and S6.

Figure 4 displays pelvis images from the rosette sequence Type 6/5 reconstructed under three different scenarios: “no correction,” “delay correction,” and “two‐parametric correction” of the sampling trajectory. The first set of images, reconstructed without any correction and using the nominal rosette trajectory only, exhibited substantial distortions and artifacts. The second set of images was obtained by applying the “delay correction” method. In this case, the quality was substantially improved, although some remaining artifacts were still present in the image, as indicated by white arrows. These residual imperfections were further suppressed by applying the “two‐parametric correction” approach, as shown in the bottom row of Figure 4. The trajectory correction improved the pelvis image quality, which could better delineate pelvic muscle contours and reproduce the fine anatomical details such as realistic structure of subcutaneous fat or the differentiation of the cortex and fatty hilum of the inguinal lymph nodes. The readers' visual artifact assessment of all three image data sets is summarized in Table 2. In the “no correction” data set, 22 images (73.33%) and 6 images (20%) were categorized as containing pronounced or highly significant artifacts (i.e., Category 4 or 5), respectively, and only 2 images (6.67%) were found to exhibit moderate artifacts (i.e., Category 3). The application of “delay correction” ensured that none of the reconstructed images was classified as Category 4 or 5 and produced the following results: Four images (13.33%) exhibited moderate artifacts, 21 images (70.0%) exhibited minor artifacts (i.e., Category 2), and 5 images (16.67%) had no artifacts (i.e., Category 1). Further improvement was achieved using “two‐parametric correction.” In this case, only 1 image (3.33%) fell into Category 3, whereas 10 images (33.33%) and 19 images (63.33%) were classified as Category 2 and Category 1, respectively.

**TABLE 2:** Summary of the artifact evaluation by two radiologists for set of images obtained by combining various rosette trajectory types and correction algorithms.

		Artifacts scores (Readers 1 and 2)								
		Reconstruction algorithm/rosette trajectory type								
		No correction			Delay correction			Two‐parametric correction		
Subject	Age (years)	6/1	6/5	6/7	6/1	6/5	6/7	6/1	6/5	6/7
1	43	5, 5	4, 4	4, 4	2, 2	2, 1	1, 1	1, 1	1, 1	2, 1
2	36	5, 5	5, 5	5, 5	3, 2	3, 2	3, 2	3, 1	2, 1	2, 1
3	29	5, 5	5, 4	5, 3	2, 2	2, 2	1, 1	1, 2	1, 1	1, 1
4	45	5, 5	5, 5	5, 4	3, 2	2, 2	2, 2	2, 1	2, 1	2, 1
5	41	5, 5	5, 4	5, 3	2, 2	2, 2	2, 2	2, 1	2, 1	2, 1
Average	38.8	4.67			1.97			1.40		

*Note*: The bottom row of the table provides information on the average subject age and the scoring for combinations of different correction algorithms and rosette trajectories. Artifacts scoring scale: 1, no artifact at all; 2, minor artifacts but without the potential to impede the assessment; 3, moderate artifacts, potentially impairing the visual assessment; 4, pronounced artifacts; 5, highly significant artifacts.

## 4 DISCUSSION

This study reports on a simple correction scheme developed for rosette imaging, which addresses the detection and correction of k‐space sampling trajectories. Rosette MRI, like many non‐Cartesian techniques, is prone to acquisition trajectory distortions. Without appropriate measures to compensate for such discrepancies, various artifacts and image degradations typically arise (Figure 2C,G,K and Figure 4 [top row]). In this study, calibration of the rosette acquisition trajectory was performed using an adapted technique previously suggested for estimation of delays in spiral MRI.^21^ However, in the case of rosette MRI, the delays estimated from k‐space zero crossings tend to form a “zigzag” pattern over the readout period (Figure 1D, Figure S3), making it difficult to properly estimate the gradient delay. We demonstrated that simple averaging of such varying delays does not always provide satisfactory results, and some residual artifacts can still be presented in the reconstructed images (Figure 2E–G and Figure 4 [middle row]).

Therefore, we hypothesized that such behavior can be explained by the full impact of the gradient channel characteristics described by the GSTF on the gradient‐waveform spectrum. These characteristics, in addition to modeling the linear phase shift responsible for time delays, also introduce alternation of the spectrum amplitudes. This consideration led us to develop a two‐parametric model that is fitted to the measured calibration delays, which we tested and experimentally verified on a structural phantom, on a $T_{2}^{*}$ relaxation measurement performed on a NnCl_2_ phantom, and on a pelvic study with a group of 5 volunteers. The conducted experiments showed that even small amplitude changes in the readout gradient, below 1% of magnitude, can cause variation in the detected delays on the order of a few microseconds (Figure 1D and Figure S3).

Considering the relatively short duration of the calibration procedure, we implemented it as a quick prescan procedure automatically performed at the beginning of each rosette sequence run. Although this study is limited to 3D stack of rosettes (i.e., Cartesian phase encoding was applied for the third slice dimension), due to the tilt slab orientation, all three physical gradient channels had to be corrected. Furthermore, in this work, we assumed that the gradient cross‐terms are much smaller than the direct terms; therefore, we did not consider their impact in this study.^31^, ^32^

Certain safety precautions related to the scanner's critical acoustic frequencies must be considered. The rosette gradient waveform contains two frequency peaks that may overlap with the scanner's critical mechanical resonance frequencies. In our study, this overlap occurred during volunteer measurements using the rosette trajectory Type 6/1, with gradient frequencies of 406 and 568 Hz. Although these frequencies fall within the scanner's critical mechanical bandwidth (centered at 590 Hz, with a bandwidth of 100 Hz, according to the manufacturer's specifications), no significant vibrations or image artifacts were observed. Nonetheless, it seems prudent to avoid pulse sequence parameters that coincide with the critical mechanical frequencies when implementing pulse sequences for routine applications.

One limitation of the study is the fact that data analyses were performed on a single slice, despite the 3D nature of the experiments (stack of rosettes). However, because the entire data volume was acquired with exactly the same readout gradient pulses, the effects of our k‐space trajectory corrections were the same for all slices. Any other discrepancies or imperfections between slices may stem from other factors, such as B_0_ inhomogeneity, transmit/receive RF coil characteristics, or even gradient coil nonlinearities.^33^, ^34^ Therefore, we limited the analysis to a single slice, as analysis of these aspects would be beyond the scope of this study.

## 5 CONCLUSIONS

We present a calibration technique that automatically adjusts the image‐reconstruction sampling grid to match the estimated k‐space trajectories for rosette imaging corrected for artifacts. The technique uses a rapid prescan calibration performed at the beginning of the sequence, which acquires information on trajectory crossings through the k‐space center. With a simple two‐parametric model reflecting the GSTF impact on the rosette gradient readout waveform, calibration scans are processed, and the actual rosette sampling trajectory is calculated. The performance and robustness of this technique were demonstrated, with both phantom experiments and pelvis imaging performed on a group of volunteers. After the initial setup in which calibration k‐space zero crossings were selected for the specific rosette trajectory types, the technique is fully autonomous without any necessary interaction from the operator. In our implementation, the prescan calibration is performed automatically with each sequence run, as the time penalty of about 3.6 s is negligible compared with the total measurement time. However, assuming high temporal stability of the gradient system, different scenarios with one‐time precalibration can also be considered. Based on our experience, we believe that the proposed calibration technique will further contribute to a broader consideration of rosette imaging for medical research applications.

## Supporting information


**FIGURE S1.** (A) Pulse sequence diagram for 3D stack‐of‐rosettes imaging and trajectory calibration. Trajectory calibration scans, conducted in the physical coordinate system, were performed at the start of the measurement (shown only for the x‐gradient channel of the positive polarity). The calibration prescans were followed by image acquisition scans performed in the logical coordinate system. (B–D) The rosette trajectory shapes prescribed by the number of oscillation (n1) and rotation (n2) cycles, which are used in this study.
**FIGURE S2.** Examples of k‐space coverage using 34 shots of the 12‐petal rosette trajectory (type 6/1) with three different golden‐ration angle increments: 137.510 (*top row*), 111.250 (*middle row*), and calculated as 300/GR ≈ 18.5410 (*bottom row*). *Left column*: Reconstructed phantom images. *Middle column*: The final k‐space coverage. *Right column*: Zoomed area, indicated by the small black rectangle in (B). The black arrows indicate the clustering of the rosette trajectories (*manifested as thick lines in the k‐space diagram*) visible in the zoomed area. The overlap of trajectories can be avoided (as seen in [I]) if angle increments for consecutive shots are calculated as the angle between two neighboring petals divided by GR. More uniform sampling minimized streaking artifact patterns in the reconstructed image (as shown in [G]).
**FIGURE S3.** Examples of calibration procedures for the 12‐petal rosette trajectory, Type 6/5 (A) and Type 6/7 (B), as measured on a phantom (refer to Figure 2) for the x‐channel of the gradient system. GR, golden ratio (approximately 1.618).
**FIGURE S4.** Example of a MnCl2 phantom image with selected regions of interest (ROIs) measuring approximately 1.6 cm^2^ and 0.4 cm^2^ and used for the relaxation study.
**FIGURE S5.** Example of in vivo pelvis rosette images acquired using the rosette acquisition trajectory Type 6/1.
**FIGURE S6.** Example of in vivo pelvis rosette images acquired with rosette acquisition trajectory Type 6/7.
**TABLE S1.** List of output parameters obtained from the trajectory calibration procedure for the structural phantom, $T_{2}^{*}$ relaxation measurements, and the pelvis study with volunteers.
