# Supplementary material for: Two‐parametric prescan calibration of gradient‐induced sampling errors for rosette MRI
Source: Magn Reson Med. 2024 Oct 22;93(3):1285–97. doi: 10.1002/mrm.30355 (PMC11680729; doi:10.1002/mrm.30355)
Supplement: Supplementary file 1 — FIGURE S1. (A) Pulse sequence diagram for 3D stack‐of‐rosettes imaging and trajectory calibration. Trajectory calibration scans, conducted in the physical coordinate system, were performed at the start of the measurement (shown only for the x‐gradient channel of the positive polarity). The calibration prescans were followed by image acquisition scans performed in the logical coordinate system. (B–D) The rosette trajectory shapes prescribed by the number of oscillation (n1) and rotation (n2) cycles, which are used in this study. FIGURE S2. Examples of k‐space coverage using 34 shots of the 12‐petal rosette trajectory (type 6/1) with three different golden‐ration angle increments: 137.510 (top row), 111.250 (middle row), and calculated as 300/GR ≈ 18.5410 (bottom row). Left column: Reconstructed phantom images. Middle column: The final k‐space coverage. Right column: Zoomed area, indicated by the small black rectangle in (B). The black arrows indicate the clustering of the rosette trajectories (manifested as thick lines in the k‐space diagram) visible in the zoomed area. The overlap of trajectories can be avoided (as seen in [I]) if angle increments for consecutive shots are calculated as the angle between two neighboring petals divided by GR. More uniform sampling minimized streaking artifact patterns in the reconstructed image (as shown in [G]). FIGURE S3. Examples of calibration procedures for the 12‐petal rosette trajectory, Type 6/5 (A) and Type 6/7 (B), as measured on a phantom (refer to Figure 2) for the x‐channel of the gradient system. GR, golden ratio (approximately 1.618). FIGURE S4. Example of a MnCl2 phantom image with selected regions of interest (ROIs) measuring approximately 1.6 cm2 and 0.4 cm2 and used for the relaxation study. FIGURE S5. Example of in vivo pelvis rosette images acquired using the rosette acquisition trajectory Type 6/1. FIGURE S6. Example of in vivo pelvis rosette images acquired with rosette acquisition trajectory Type 6/7. TABLE S1 [file MRM-93-1285-s001.pdf]

Two-parametric calibration of gradient induced sampling errors for rosette MRI.

Supporting Information

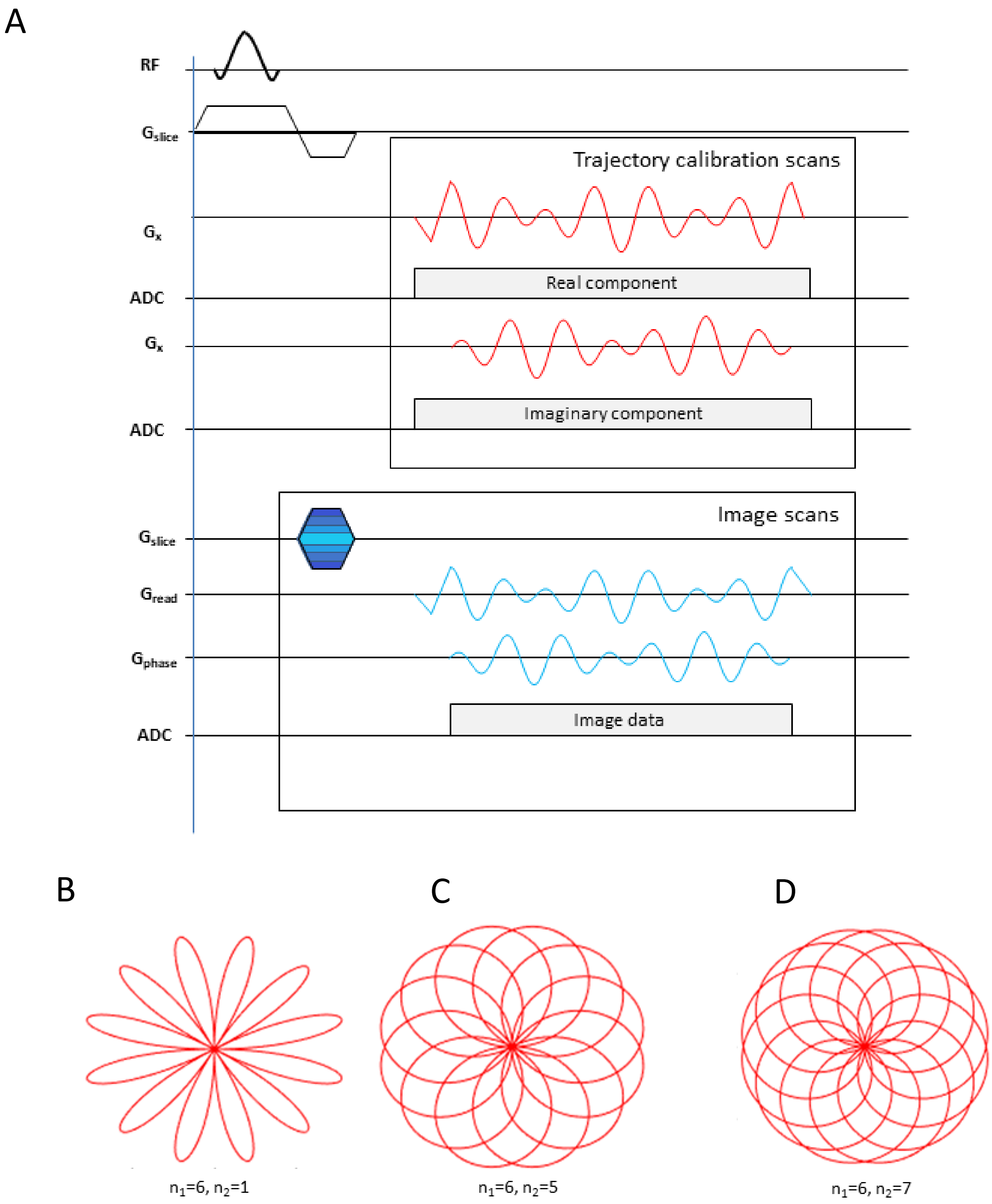

Supporting Information Figure S1: (A) Pulse sequence diagram for 3D stack of rosettes imaging and trajectory calibration. Trajectory calibration scans, conducted in the physical coordinate system, were performed at the start of the measurement (shown only for the x-gradient channel of the positive polarity). The calibration pre-scans were followed by image acquisition scans performed in the logical coordinate system. The rosette trajectory shapes prescribed by the number of oscillation (n1) and rotation (n2) cycles, which are used in this study, are shown in (B, C, and D).

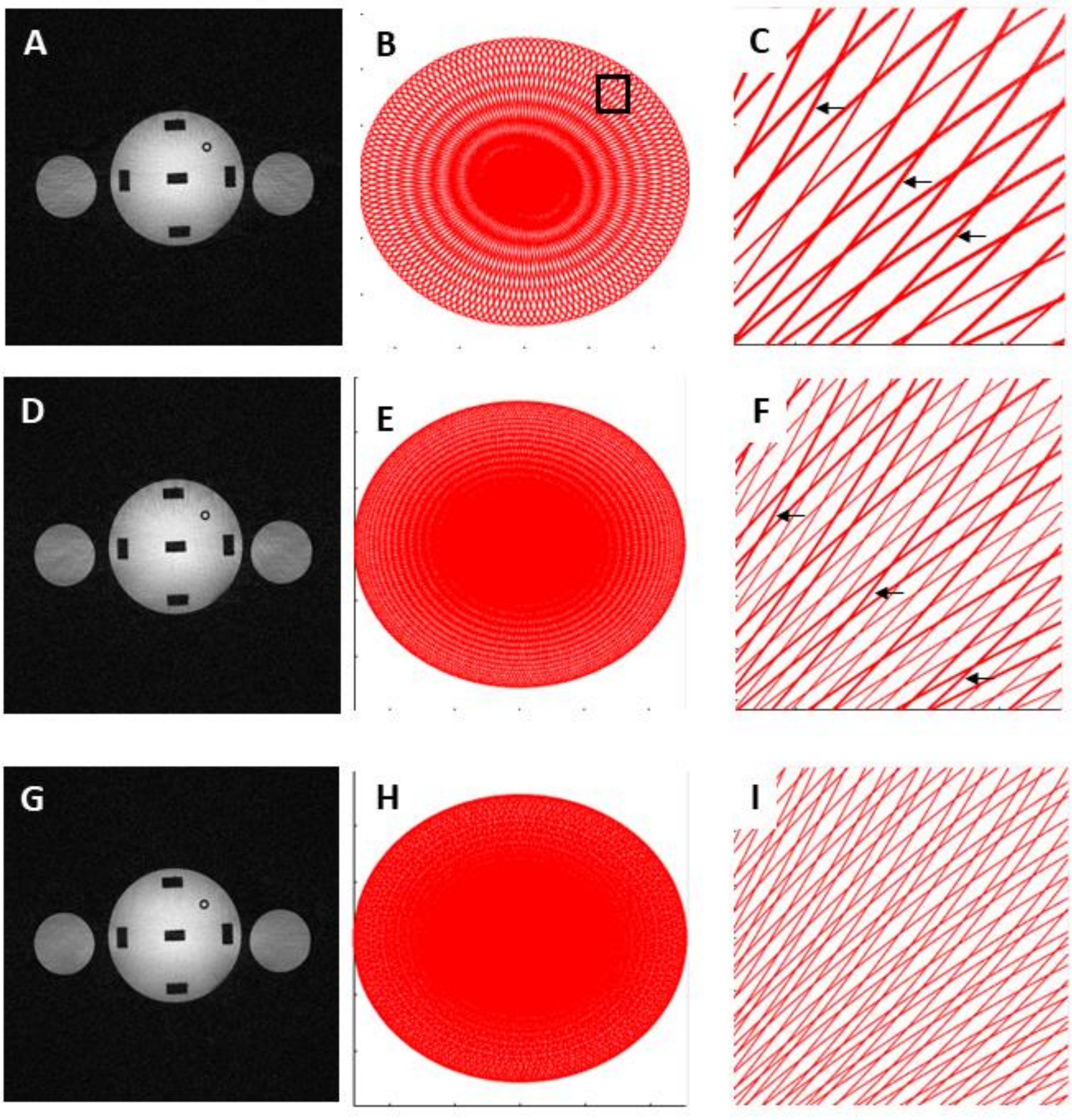

Supporting Information Figure S2: Examples of k-space coverage using 34 shots of the 12-petal rosette trajectory (type 6/1) with three different golden-ration angle increments: 137.51^0 (top row), 111.25^0 (middle row), and calculated as 30^0/GR ≈ 18.541^0 (bottom row), where GR stands for the golden ratio (approximately 1.618). The left column shows reconstructed phantom images, the middle column represents the final k-space coverage, and the zoomed area (indicated by the small black rectangle in B) is shown in the right column. The black arrows indicate the clustering of the rosette trajectories (manifested as thick lines in the k-space diagram) visible in the zoomed area. The overlap of trajectories can be avoided (as seen in I) if angle increments for consecutive shots are calculated as the angle between two neighboring petals divided by GR. More uniform sampling minimized streaking artifact patterns in the reconstructed image (as shown in G).

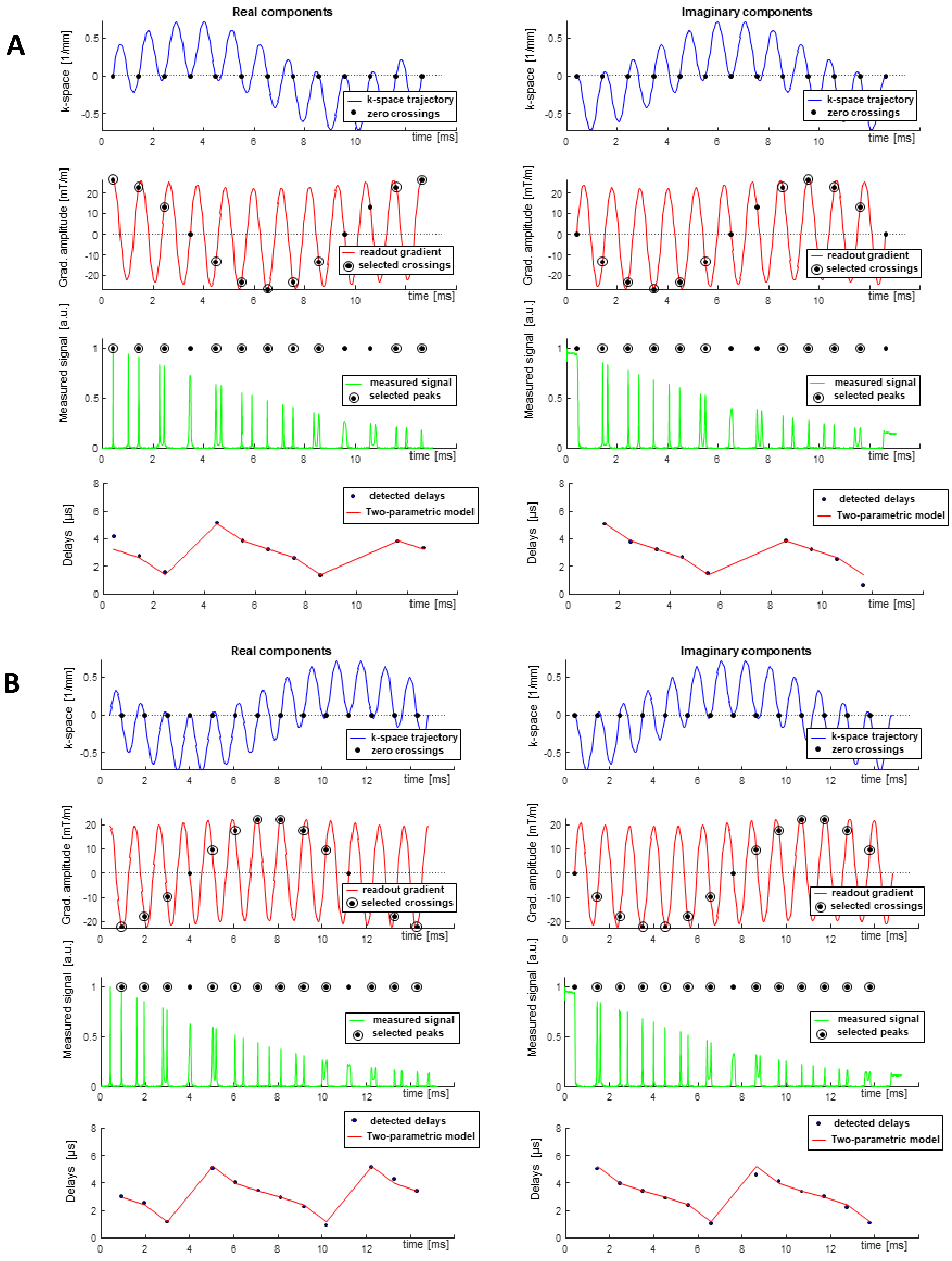

Supporting Information Figure S3: Examples of calibration procedures for the 12-petal rosette trajectory, type 6/5 (A) and type 6/7 (B), as measured on a phantom (refer to Fig. 2) for the x-channel of the gradient system

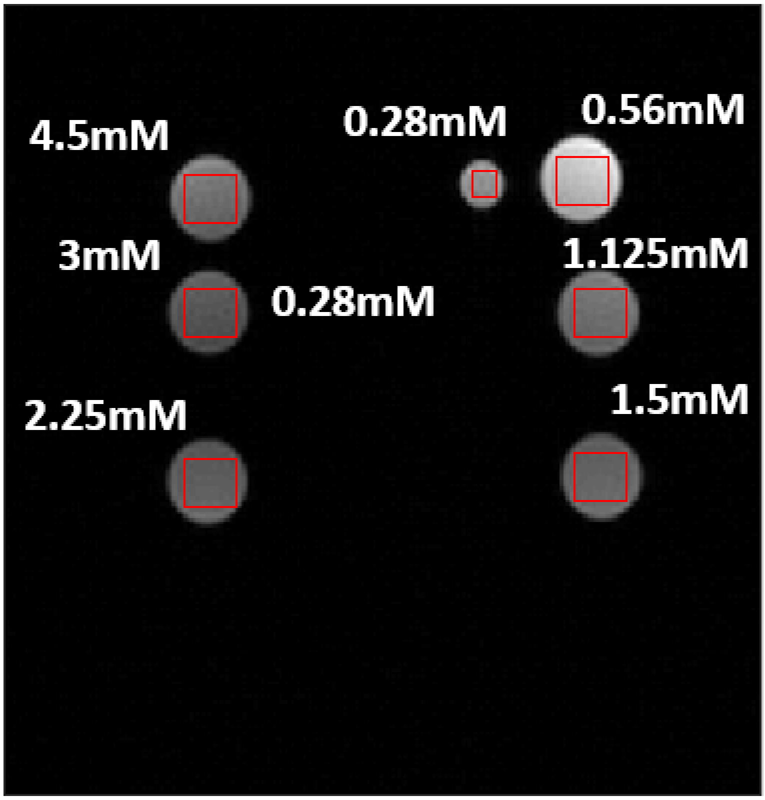

Supporting Information Figure S4: Example of a $MnCl_2$ phantom image with selected regions of interest (ROIs) measuring approximately 1.6 cm^2 and 0.4 cm^2 and used for the relaxation study.

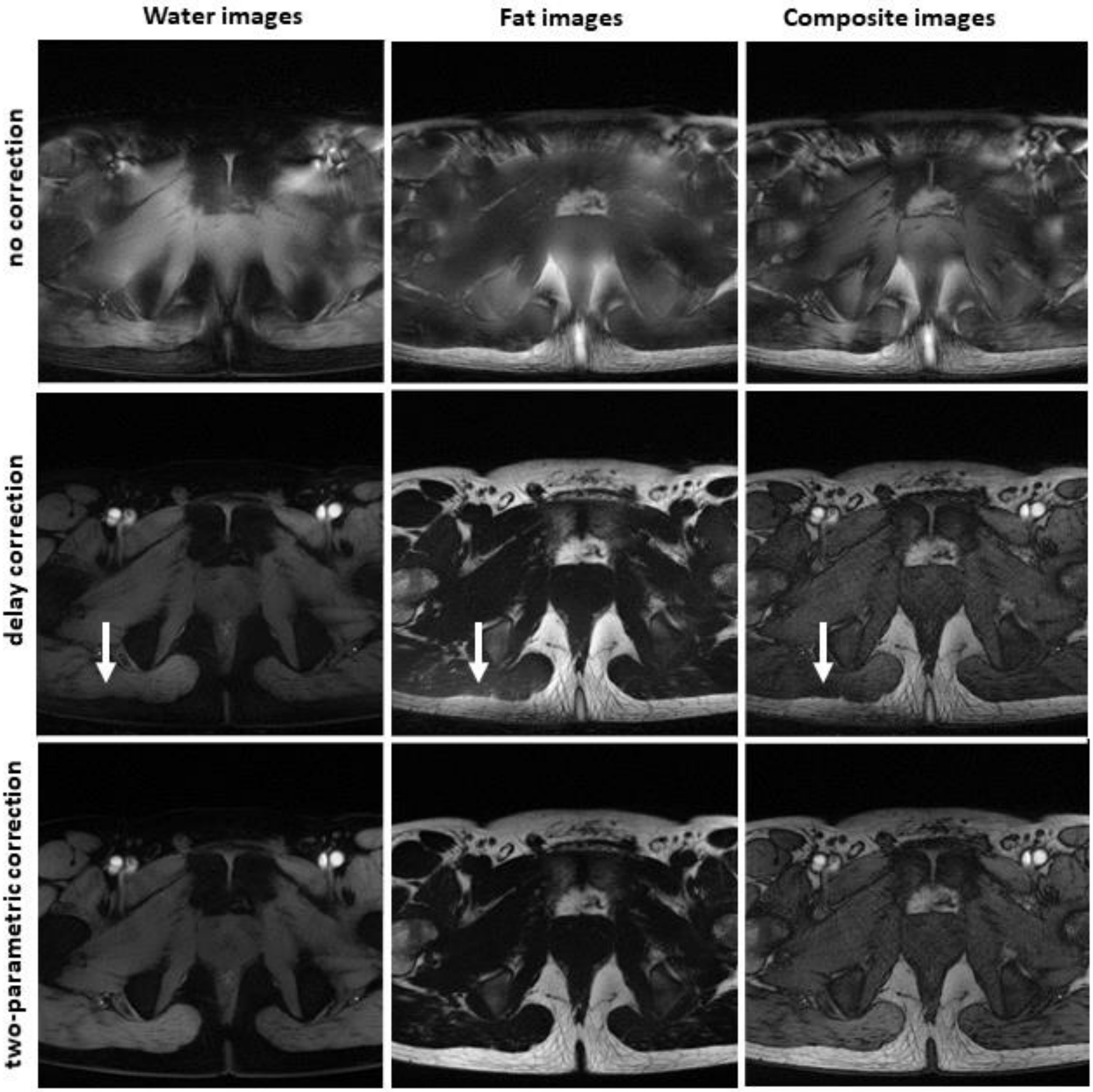

Supporting Information Figure S5: Example of in-vivo pelvis rosette images acquired using the rosette acquisition trajectory type 6/1.

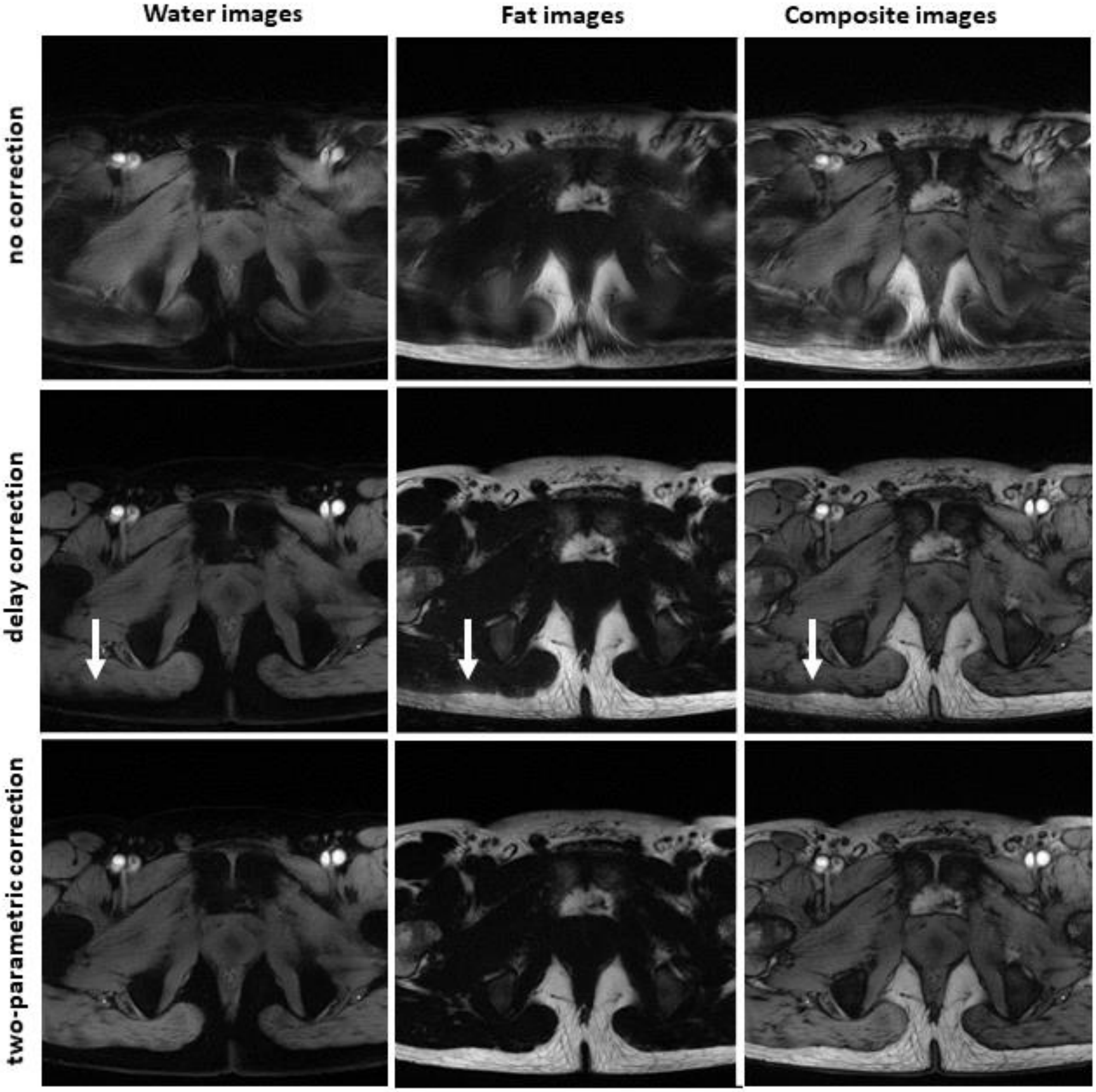

Supporting Information Figure S6: Example of in-vivo pelvis rosette images acquired with rosette acquisition trajectory type 6/7.

Gradient channel	Rosette trajectory type / correction method								
	6/1			6/5			6/7		
	two-parametric correction		delay correction	two-parametric correction		delay correction	two-parametric correction		delay correction
	delay (us)	R	delay (us)	delay (us)	R	delay (us)	delay (us)	R	delay (us)
**Structural phantom**									
X	3.684	0.998	3.705	3.125	0.993	2.903	3.178	0.992	3.206
Y	4.405	0.999	4.426	3.894	0.993	3.541	3.871	0.991	4.228
Z	4.750	1.001	4.785	4.792	1.003	4.771	4.810	1.001	4.786
**Relaxation T_2^* phantom**									
X	3.825	0.998	4.655	3.601	0.994	3.828	3.607	0.994	4.342
Y	4.443	0.999	4.644	4.295	0.996	4.355	4.098	0.996	4.404
Z	4.864	1.000	4.864	4. 981	1.001	4.896	4.622	1.000	4.781
**Volunteer #1**									
X	3.640	0.998	3.795	3.393	0.996	3.494	3.414	0.995	3.686
Y	4.390	0.999	4.509	4.119	0.996	4.059	4.161	0.995	4.304
Z	4.573	1.001	4.608	4.635	1.003	4.737	4.636	1.002	4.710
**Volunteer #2**									
X	3.584	0.998	3.782	3.385	0.996	3.340	3.372	0.995	3.760
Y	4.369	0.999	4.407	4.139	0.995	4.054	4.164	0.995	4.447
Z	4.604	1.001	4.757	4.813	1.002	4.931	4.795	1.002	4.956
**Volunteer #3**									
X	3.583	0.999	3.610	3.383	0.995	3.321	3.362	0.995	3.546
Y	4.409	0.999	4.436	4.190	0.995	3.973	4.141	0.995	4.280
Z	4.723	1.001	4.760	4.781	1.003	4.899	4.878	1.001	4.970
**Volunteer #4**									
X	3.583	0.999	3.620	3.392	0.995	3.291	3.455	0.995	3.580
Y	4.393	0.999	4.474	4.166	0.995	4.025	4.136	0.995	4.305
Z	4.772	1.001	4.791	4.802	1.003	4.910	4.826	1.002	4.907
**Volunteer #5**									
X	3.629	0.998	3.658	3.352	0.995	3.368	3.401	0.995	3.595
Y	4.355	0.999	4.511	4.145	0.995	4.069	4.166	0.995	4.376
Z	4.693	1.001	4.707	4.753	1.003	4.844	4.762	1.002	4.807

Supporting Information Table S1 List of output parameters obtained from the trajectory calibration procedure for the structural phantom, T_2^* relaxation measurements, and the pelvis study with volunteers.
